# Effects of Chicory/Perennial Ryegrass Swards Compared with Perennial Ryegrass Swards on the Performance and Carcass Quality of Grazing Beef Steers

**DOI:** 10.1371/journal.pone.0086259

**Published:** 2014-01-28

**Authors:** Christina L. Marley, Rhun Fychan, John W. Davies, Nigel D. Scollan, R. Ian Richardson, Vince J. Theobald, Elizabeth Genever, Andy B. Forbes, Ruth Sanderson

**Affiliations:** 1 Animal and Microbial Sciences, Institute of Biological, Environmental and Rural Sciences (IBERS), Aberystwyth University, Gogerddan, Ceredigion, United Kingdom; 2 University of Bristol, Food Science and Food Safety Group, Division of Farm Animal Science (DFAS), Langford, Bristol, United Kingdom; 3 EBLEX, Agriculture and Horticulture Development Board, Stoneleigh Park, Kenilworth, Warwickshire, United Kingdom; 4 Merial Animal Health, Lyon, France; Wageningen UR Livestock Research, Netherlands

## Abstract

An experiment investigated whether the inclusion of chicory (*Cichorium intybus*) in swards grazed by beef steers altered their performance, carcass characteristics or parasitism when compared to steers grazing perennial ryegrass (*Lolium perenne*). Triplicate 2-ha plots were established with a chicory/ryegrass mix or ryegrass control. Forty-eight Belgian Blue-cross steers were used in the first grazing season and a core group (*n* = 36) were retained for finishing in the second grazing season. The experiment comprised of a standardisation and measurement period. During standardisation, steers grazed a ryegrass/white clover pasture as one group. Animals were allocated to treatment on the basis of liveweight, body condition and faecal egg counts (FEC) determined 7 days prior to the measurement period. The measurement period ran from 25 May until 28 September 2010 and 12 April until 11 October 2011in the first and second grazing year. Steers were weighed every 14 days at pasture or 28 days during housing. In the first grazing year, faecal samples were collected for FEC and parasite cultures. At the end of the first grazing year, individual blood samples were taken to determine *O. ostertagi* antibody and plasma pepsinogen levels. During winter, animals were housed as one group and fed silage. In the second grazing year, steers were slaughtered when deemed to reach fat class 3. Data on steer performance showed no differences in daily live-weight gain which averaged 1.04 kg/day. The conformation, fat grade and killing out proportion of beef steers grazing chicory/ryegrass or ryegrass were not found to differ. No differences in FEC, *O. ostertagi* antibody or plasma pepsinogen levels of beef steers grazing either chicory/ryegrass or ryegrass were observed. Overall, there were no detrimental effects of including chicory in swards grazed by beef cattle on their performance, carcass characteristics or helminth parasitism, when compared with steers grazing ryegrass.

## Introduction

Chicory (*Cichorium intybus*) is a perennial deep-rooting broad-leafed forage herb of the Asteraceae which has been regarded worldwide as a valuable constituent of pastures for grazing livestock for many years [1]–[2]. Since the development of more modern commercial cultivars from the 1980s onwards [3]–[4], the use of chicory has been steadily increasing, albeit predominately in sheep and deer production systems [5]. Agronomically, chicory is highly productive [6], has a high feed value [7] and has been found to improve pasture quality by improving the seasonal availability of high quality forage [8]. Chicory has a variable crude protein (CP) concentration (ranging between 150–260 g kg dry matter (DM)^−1^), depending on nitrogen input [6], with some indication that it is more efficient at capturing soil nitrogen compared to perennial ryegrass (*Lolium perenne*) when grown under the same conditions [8]. Research has also shown that chicory contains a higher water-soluble carbohydrate concentration [9]–[10] and a higher mineral and trace element concentration than ryegrass [8], [11]–[13].

Renewed interest in this forage crop has been supported by findings from research confirming the attributes for this forage when fed to grazing livestock. These include its ability to increase the productivity of finishing lambs [14]–[15] and red deer [16]–[17], to reduce rumen nitrogen losses [5], to reduce internal parasites [18] and to improve carcase conformation in finished lambs [19] when compared to ryegrass. However, despite this, there has been relatively little research into the effects of this forage when utilised within beef production systems [20]–[23], with none of these studies investigating the effects of chicory on internal nematode parasites in beef animals. Research studying the performance of bull beef over two short-term periods when grazing chicory [20] concluded that chicory was able to support maximum live-weight gains for 6–8 month old calves. In 1990, research [21] with chicory grazed in a short-term experiment with calves and bulls and concluded that further studies were needed to determine the production response of beef cattle grazed on chicory maintained in vegetative state. Other research into the effects of beef grazing chicory on bite rate [22] concluded that the uniform quality of chicory swards supported a hypothesis that this forage could be useful for livestock having high nutrient and DM intake requirements. Chicory was able to produce live-weight gains in beef cattle that were comparable to those for beef produced from pastures sown with an annual ryegrass (*Lolium multiflorum*) which is typically grown in the southeast of the United States [23]. In more recent work, steers grazing chicory had higher live-weight gains than those grazing bermudagrass (*Cynodon dactylon*), cowpea (*Vigna unguiculata*) or pearl millet (*Pennisetum glacum*) but not those grazing lucerne (*Medicago sativa*) [24]. However, there have been no studies into the effects of this forage when compared to perennial ryegrass swards over two grazing seasons within a temperate forage-based beef finishing system.

The aim of this experiment was to determine the effects of chicory/perennial ryegrass swards compared with perennial ryegrass swards on the productivity, helminth parasitism and carcass quality of grazing beef steers over a two year production period.

## Materials and Methods

### Ethical Statement

All animal procedures followed strict guidelines as set forward in the Animals (Scientific Procedures) Act (1986) and approved by the Home Office (HO), UK and were performed under the HO licence no. PPL40/3166. Animals were certified as not suffering any adverse effects as a result of any procedures at the end of the experimental period by the named HO veterinary officer.

### Experimental Approach

During the establishment year (2009), replicate grazing plots were cultivated and sward cover and quality was achieved and maintained by rotational grazing management with sheep. The beef experiment was conducted over the following two consecutive grazing seasons, with individual animals remaining on the same replicate forage plot in both years. In the first grazing year (2010), the experiment focussed on the performance of the steers but also monitored any treatment effects on their internal gastro-intestinal (GIT) helminth parasites during their first grazing season. In the winter period between 2010 and 2011, animals were kept as one group and offered a standard diet of ryegrass silage plus straw. In the second grazing year (2011), measurements determined the performance and the carcass characteristics of beef steers at finishing.

### Experimental site and treatments

#### Forage establishment

Triplicate 2 ha field plots were established with either a chicory (cv. Puna II)/perennial ryegrass (cv. Premium) mix or a perennial ryegrass control (cv. Premium) at Penglais farm, Aberystwyth University, Wales, United Kingdom (52°25′46′′N 4°4′13′′W). The experimental area was treated with glyphosate herbicide at 4 litre ha^−1^ before ploughing. Lime, phosphate and potash were applied to correct any soil deficiencies prior to cultivation. Nitrogen was applied at a rate of 67 kg ha^−1^ to the seed bed. Perennial ryegrass plots were sown at a rate of 30 kg ha^−1^ and chicory/ryegrass plots were sown at a rate of 7.5 kg ha^−1^ chicory and 22.5 kg ha^−1^ perennial ryegrass using an Einbock harrow/seeder on 4 and 5^th^ June 2009. Sowing depth for both treatments was 10 mm. All areas were then rolled with a flat roller. On 9 July, one chicory/ryegrass and one ryegrass replicate plot was topped using a flail mower to reduce the unsown species present at that time in the establishing swards. Slug pellets were applied at a rate of 2.5 kg ha^−1^ to all plots on the 10 July 2009. A total of 709 weaned lambs grazed the plots to maintain herbage quality during the establishment year. Lambs rotationally grazed each plot from approximately 6 weeks post-cultivation (17 July) until 11 September, when the average sward height of the experimental swards was recorded as 5 cm.

#### Animals

Forty-eight Belgian Blue – dairy cross steers (mean ± s.e.m. liveweight: 185±4.0 kg; aged approximately 7 months on Day 0) were used for the experiment. In the first grazing season (2010), eight animals grazed each replicate plot (*n* = 24 per treatment) to match forage biomass availability and to determine the effects of chicory/ryegrass compared with ryegrass on GIT parasites in steers. A core group of 36 animals (mean ± s.e.m. liveweight: 184±4.7 kg on Day 0) were retained for the whole two year production and finishing period, so there were 6 (from the initial 8) animals grazing each replicate plot in the second grazing season (2011) (*n* = 18 per treatment). All steers were sourced in February 2010, housed and offered a first cut ryegrass silage plus 2 kg head^−1^ of a standard commercial beef fattening concentrate ration. Fourteen days prior to turn-out, the amount of concentrates offered was gradually reduced to allow for rumen adaptation. All steers received two doses, one month apart, of a lungworm vaccine (Huskvac™, MSD Animal Health, Milton Keynes, UK) treatment prior to turn-out.

#### Plot Management

During both grazing years, the experimental plots were divided using an electric fence and managed as two separate halves (sub-plot A and B) of 1 ha. Each half was rotationally-grazed and cut for silage as required to maintain herbage availability and quality throughout the grazing season. During the first grazing season, sub-plots were further sub-divided into two halves. A silage cut was taken from all plots immediately prior to Day 0 (18 May), with a second silage cut being taken from sub-plot A and B on 5 July 2010 and 9 August, respectively. The management of each plot was alternated so that both halves had the same number of grazing days and silage cuts by the end of the first grazing season. Inorganic N was applied at 200 kg N ha^−1^ per annum, split across 5 dates at rates of 60, 40, 40, 30 and 30 kg N ha^−1^ for approx. mid-March, mid-May, early June, early July and early August, respectively.

### Measurements

#### Forages

The number of chicory plants within a 360 mm×250 mm quadrat at 6 random sites across each plot was counted in spring (or 6–8 weeks post-establishment in 2009) and autumn each year and the plant population per m^2^ calculated. The germination index of the seed of each forage was determined by placing approximately 200 seeds on dampened tissue in a seed tray at 20°C for 14 days. Sward height was recorded fortnightly throughout the grazing period as a management tool by taking 40 measurements, either per plot or from the area available to the steers, of the unextended sward height using a Hill Farm Research Organisation sward stick whilst walking in a ‘W’ transect across the measurement area [25]. Herbage availability was determined from six 0.5×1 m quadrats, cut to ground level, within each sub-plot at the start and end of grazing each rotationally-grazed area. The fresh weight of each sample was determined and a 400 g sub-sample taken to determine DM content. A second 400 g sub-sample was taken from each sample, and bulked on a plot basis. This material was thoroughly mixed and a 100 g sub-sample taken for botanical separations. The fresh forage was separated when fresh into sown and unsown species (clover, broad-leaf weeds and weed grasses) the separated material dried, and the composition of the sward calculated on a DM basis. A second 100 g sub-sample of the bulked material from each sub-plot was freeze dried and submitted for chemical analysis to determine ash, water-soluble carbohydrates (WSC), crude protein (CP), and neutral-detergent fibre (NDF) concentrations. The DM contents of the forages were determined by drying to constant weight at 100°C in a forced draught oven, and the DM content of the samples for chemical analysis determined by freeze-drying. Ash concentrations were measured by igniting samples in a muffle furnace at 550°C for 16 h. Total nitrogen (TN) concentrations were determined using a Leco FP 428 nitrogen analyser (Leco Corporation, St. Joseph, MI, US) and expressed as CP (TN×6.25). NDF analysis was carried out according to the methods given in [26]. *In vitro* Digestible Organic Matter in the total Dry matter (DOMD) was predicted by the pepsin cellulase method [27]. Concentrations of WSC were determined spectrophotometrically using anthrone in sulphuric acid on a Technicon Autoanalyser [28].

#### Animals

The experiment comprised of a standardisation and a measurement period. During the standardisation period of 28 days [29], steers grazed a ryegrass/white clover permanent pasture adjacent to the main experimental plots as one group. Liveweight data collected on Day minus 28, Day minus 14 and Day 0 were used to determine covariate growth rates. Animals were allocated to their respective treatment on the basis of live weight, body condition score (BCS) and faecal egg counts (FEC) determined 7 d prior to the measurement period (Day minus 7). In 2010, the core 36 animals were balanced across replicate plots as well as the experiment being balanced for the 48 animals that grazed the plots that year. On Day 0, steers were placed into treatments × replicate groups, and placed on experimental plots sown with either a chicory/ryegrass or a ryegrass control. Individual steers were weighed and body condition scored at the start of the measurement period and then every 14 d. Body condition score (BCS) was determined by the same person on each occasion. The measurement period started on 25 May 2010 (Day 0) and 12 April 2011 (Day 322) in the first and second grazing year, respectively, and continued until herbage availability dictated the end of grazing on 28 September 2010 (Day 126) and 11 October 2011 (Day 504), respectively.

#### Parasite control, anthelmintic treatment and monitoring

In the first grazing season, parasite levels of all 48 (n = 8 per replicate) animals, alongside live-weight gains and BCS, were monitored whilst following a regular anthelmintic treatment to control gastro-intestinal nematode parasites. The interval between anthelmintic treatments allowed for a good level of parasite control in this class of animal [30]–[31] and included between 21–28 days of parasite monitoring following the 28 days of persistent anthelmintic activity from the treatment. Faecal samples were collected on Day 0, 28, 70, 84 and 126 for faecal egg count (FEC) and parasite culture determinations. Faecal samples for FEC were taken immediately prior to any routine anthelmintic treatment (Eprinex Pour-on, 0.5% eprinomectin (Merial Animal Health, Harlow, Essex, UK) at a rate of 1 ml per 10 kg liveweight, given on Day 28, 84 and 126. FEC and culture samples were submitted immediately to the Parasitology Department, VLA Laboratories, Aberystwyth, UK. FEC were determined using a modified McMaster technique [32], with 1 egg representing 50 eggs g^−1^ of fresh faeces. Faecal cultures of *Trichostrongyle* type eggs to third stage larvae (L3) consisted of a 10 g faecal sample per individual steer, bulked per plot and incubated at 27°C±3°C for 7 days. Faecal DM was determined by placing a 15 g sample of faeces at 95°C for 48 h. On day 126 (prior to winter housing), a blood sample was taken from each animal to determine *O. ostertagi* antibody levels using an enzyme-linked immuno-assay (ELISA), with results expressed as an optical density ratio (ODR) [33]. Plasma pepsinogen levels were also measured in the blood sample using a colorimetric method, with enzyme activity expressed as units (U) of tyrosine [34], based on the method described in [35]. Blood samples were taken into vacutainers without heparin and the blood was left to clot (approx. 30 min) before samples were centrifuged at 1300 g for 10 min. The serum was then stored frozen at −20°C prior to analysis.

#### Winter housing period

From 28 September 2010 until 12 April 2011, all animals were housed as one group and offered first cut clamp grass silage *ad libitum* mixed with barley straw offered at 0.5 kg (fresh weight) head^−1^ per day^−1^. The nutritional value of the silage was determined by NIRS analysis and is shown in **Table 1**. The live weight of the steers was determined every 28 d during housing. At the end of housing, animals returned to their respective replicate grazing plots and their performance was recorded every 14 d, as during the first grazing season.

**Table 1:** Silage composition of standard ryegrass clamp silage offered to steers during winter housing period (winter 2010–2011).

Chemical Composition	g/kg DM (unless otherwise stated)
Dry Matter (g/kg fresh weight)	448
Ash	7.7
Neutral-detergent Fibre	435
Metabolisable Energy (MJ/kg DM)	11.4
Crude Protein	135
pH	4.4
Ammonia N (g/kg total N)	44
Lactic Acid	40.6
Volatile Fatty Acids	20.2

#### Carcass characteristics

In the second grazing year, steers were selected for slaughter when they were deemed as having reached a target fat class of 3, with a target conformation of U, and their days to finish was recorded. All carcasses were commercially graded for conformation (EUROP classification) and fat class. Live weight and cold carcass weight were used to determine killing-out percentages.

### Data processing and statistical analysis

Effects of sown forage on pasture characteristics, cattle performance and parasitism data were examined by analysis of variance (ANOVA) of the completely randomised design using Genstat® Version 14.2 [36]. Live-weight gain within each experimental period was calculated by Theil regression [37] of fortnightly live weights. Initial live weight (day 0) was used as a covariate in the analysis of live-weight gain during the first grazing season and live-weight gain during the winter and second grazing season were adjusted for live-weight gain in their respective preceding period. Since the growth rates within each experimental period varied, the overall live-weight gain was calculated as (final – Day 0 liveweight)/days to finish and data analysed by ANOVA, with Day 0 live-weight used as a co-variate. Total faecal egg count data were adjusted for faecal DM content and transformed (to the square root of y+1) before the forage treatments were compared at each sampling point using the previous sample values as the covariate. Carcass slaughter grades were converted to numeric score prior to analysis as described in [38] prior to statistical analysis by ANOVA.

## Results

### Forages

#### Germination percentages and chicory plant populations

The results of the germination test showed that the chicory and ryegrass seed had a germination success of 89 and 95%, respectively. Plant population data showed that chicory established well in all three replicate plots. Chicory plant numbers of 110–131 plants per square metre were recorded during the period from spring 2010 until autumn 2011, when the population declined to 56 plants m^−2^.

#### Sward composition

Botanical composition data showed that chicory/ryegrass swards contained 24 and 14% chicory on a DM basis in the first and second grazing year, respectively. The ryegrass plots had a higher forage biomass available to steers in both grazing seasons, when determined on a DM basis. The biomass of perennial ryegrass available to the steers in the chicory swards was consistent between years, and changes observed in botanical composition were due to a reduction in the chicory yield and an increase in introgressed weed grasses, white clover and broadleaf weeds. Despite sward heights of all plots being maintained at the same height, there was a higher biomass of available forage on ryegrass only plots in the first (*P<*0.05) but not the second grazing season. Ryegrass only swards were found to have a higher WSC concentration in the first grazing season and higher fibre concentrations in the second grazing season. The DM of forage on ryegrass plots was significantly higher in both the first (*P<*0.05) and second (*P<*0.01) grazing season. The quality of the ryegrass silage offered during the winter housing period had a high DM and ME content and a moderate crude protein concentration compared to an average ryegrass silage (**Table 2**).

**Table 2:** Mean forage biomass, botanical composition, sward height, forage DM and chemical composition of grazed plots within each grazing season (2010 (*n* = 11) and 2011 (*n* = 14)).

	First grazing season (2010)					Second grazing season (2011)		
	Ryegrass	Chicory/ryegrass	sed	*P*	Grass	Chicory/ryegrass	sed	*P*
Forage biomass (kg DM/ha)	1631	1313	71.8	*	1388	1280	86.5	ns
Perennial Ryegrass (kg DM/ha)	1602	971	126.4	**	1261	1007	101.2	ns
Chicory (kg DM/ha)	-	324	-	-		174	-	-
Weed Grass (kg DM/ha)	14.0	7.5	23.8	ns	47.8	29.2	22.6	ns
White Clover (kg DM/ha)	3.2	2.3	5.0	ns	9.7	8.4	3.39	ns
Broadleaf weeds (kg DM/ha)	11.5	7.5	5.7	ns	31.6	30.7	19.7	ns
Chicory %	-	24	-	-	-	14		
Sward Height (cm)	13.4	12.7	0.39	ns	12.1	11.7	0.47	ns
Dry Matter (g/kg fresh matter)	209	184	5.4	*	229	209	2.71	**
Crude Protein (g/kg DM)	157	168	5.3	ns	148	157	5.34	ns
WSC (g/kg DM)	128	107	7.7	*	169	154	6.43	ns
NDF (g/kg DM)	531	471	25.2	ns	525	495	9.92	*
Ash (g/kg DM)	96	123	17.0	ns	80	89	4.35	ns

WSC, water-soluble carbohydrates; NDF, neutral-detergent fibre; ns, not significant; *, *P*<0.05; **, *P<0.01*.

### Animals

#### Performance

The liveweight data showed that there were no differences between grazing treatments in the performance of beef steers during either the first or second grazing season and this was reflected in the number of days to slaughter (**Table 3**). Data on steer performance within each of the three phases of the experiment (the first grazing season, winter housing period and then second grazing season) and the overall live-weight gain showed that there were no differences in the daily live-weight gain of steers grazing chicory/ryegrass or ryegrass only swards in this experiment.

**Table 3:** Performance of beef steers (*n* = 36) grazing either chicory/ryegrass or ryegrass only swards during their first and second grazing season and during the winter period between whilst housed.

	Ryegrass	Chicory/ryegrass	sed	*P*
Live-weight gain (kg/day)				
First grazing season	1.15	1.09	0.052	ns
Winter period	1.08	1.11	0.049	ns
Second grazing season Overall (Day 0 to finish)	1.07 1.01	1.00 0.98	0.135 0.033	Ns ns
Days to slaughter^a^	137	136	12.7	ns

a , number of days from turnout in Year 2 to slaughter.

#### Parasite status

The results of the gastro-intestinal parasite data are shown in **Table 4**. There were no differences in faecal egg count (FEC), faecal DM or DM-adjusted FEC, *O. ostertagi* antibody or plasma pepsinogen levels of beef steers grazing either chicory/ryegrass swards or ryegrass only swards. These data showed that the steers experienced a moderate challenge of parasitic nematodes over the grazing season. Faecal cultures indicated that *Ostertagia ostertagi* and *Cooperia* spp. were the main parasite species present in the steers. As a proportion of the total number of larvae present in the cultures, there were between 0.48–0.75 *O. ostertagi* and 0.52–0.25 *Cooperia* spp., respectively, found in faecal cultures across the grazing season, with only negligible levels of *Trichostrongylus* species being found on Day 84 (0.02) and Day 126 (0.04). Plasma pepsinogen levels also indicate that the steers were challenged with *O. ostertagia*. The different parasite species were present in the same proportions in faeces (within 0.01–0.02) from both treatments on each sampling date.

**Table 4:** Faecal egg count (g/DM) (square root transformed), *O. ostertagi* antibody (as Optical Density Ratio) or plasma pepsinogen (expressed as units of tyrosine (U)) levels of beef steers (*n* = 48) in their first grazing season.

	Ryegrass	Chicory/ ryegrass	sed	*P*
Faecal egg count				
Day 0	51.7	51.4	4.25	ns
Day 28	65.0	65.2	7.40	ns
Day 70	25.9	31.2	2.70	ns
Day 84	37.2	45.6	3.94	ns
Day 126	20.8	23.1	7.64	ns
*O. ostertagi* antibody	0.72	0.70	0.06	ns
Plasma Pepsinogen	2.15	2.07	0.197	ns

ns, not significant.

#### Carcass characteristics

The conformation, fat grade, killing out proportion and carcass weight of beef steers grazing ryegrass or ryegrass/chicory swards were not found to differ in this study (**Table 5**). The conformation grades in Table 5 are equivalent to grade R. The carcass grades showed that the fat class was as targeted (at class 3) and therefore did not differ between treatments. There were no differences in the carcass characteristics of beef steers grazing either chicory/ryegrass swards or ryegrass only swards.

**Table 5:** Carcass characteristics of beef steers (*n* = 36) grazing either chicory/ryegrass or ryegrass swards.

	Ryegrass	Chicory/ryegrass	sed	*P*
Conformation	85.0	92.8	8.74	ns
Fat grade	52.8	61.2	6.57	ns
Slaughter weight	638	632	12.3	ns
Killing out	0.55	0.56	0.004	ns
Carcass Weight				
Right side hot	176.8	178.1	3.21	ns
Right side cold	174.1	175.6	3.23	ns
Total cold	350.9	353.7	6.44	ns

ns, not significant.

## Discussion

### Forages

#### Germination percentages and chicory plant populations

Plant populations showed that chicory established well in all field plots, as supported by the high germination success recorded for the seeds in the laboratory. Plant population and forage botanical composition data confirmed visual observations that the chicory/ryegrass grazing plots were sufficiently chicory-rich to adequately test the effects of chicory within a pasture-based beef finishing system. It should be noted that although the DM content of the sward was 24 and 14 per cent in the first and second grazing season, respectively, visibly the chicory/ryegrass swards appeared more chicory-dense than this data would indicate, due to the low DM content of the chicory. It is recommended that chicory pastures should be reseeded if the chicory plant population falls below 25 plants per metre squared [7], whereas plant populations were still at twice this level when the current study was completed in autumn 2011.

#### Sward composition

Pasture management using a rotational grazing system was shown to maintain adequate herbage biomass and sward heights to ensure that forage availability did not restricted the voluntary intake capacity of the steers at any time point during this study. Chicory was found to be highly productive over the grazing season despite being virtually dormant in winter, as shown in previous studies [39]. However, it should be noted that during the second grazing year, there was a need to top the chicory plots using a flail mower post –grazing on three occasions to maintain the swards in a vegetative state. This was due to a combination of a tendency for the chicory plants to bolt during dry/high growth periods and the need for the stocking rate to be kept constant for the purposes of the experiment. This finding is in agreement with other research [40] showing that grazing management affected the production of reproductive stems in chicory, in addition to changes in water supply. It was deemed essential to maintain all grazing swards in a similar vegetative state to provide a direct experimental comparison as otherwise, forage mass and forage quality, as affected by sward height [41], leaf to stem ratio and sward structure [42] could have affected the voluntary intake of chicory by the grazing beef steers. In summary, the plant populations and forage data confirm that the establishment and performance of the forages in the present study can be deemed as representative of good agricultural pastures, containing relatively low levels of unsown species and maintained in a vegetative state.

The DM content of the chicory/ryegrass swards were lower than the ryegrass treatment, reflecting other research findings for a low DM content of chicory in comparison to ryegrass [9], [43]and that increasing proportions of chicory within ryegrass swards reduced the DM content of the harvested forage [44]. Factors affecting voluntary intake in ruminants are complex in nature but it is influenced by forage DM content [45] and it was possible that the low DM of chicory could have reduced voluntary intake, and thus the performance, of the beef steers in the current study. However, the inclusion of chicory increased the CP concentration of the sward by, on average, ten percent in this study. Chicory has a variable CP concentration depending on nitrogen input and, typically, it has a higher CP than ryegrass [46], even when grown under the same conditions [8], with increasing proportions of chicory within ryegrass swards having been shown to increase the CP content of the forage harvested [44]. As the CP concentration of forages, when fresh or ensiled, has been shown to increase voluntary intake in ruminants [47]–[48], this may have negated the effects of the lower DM content of chicory on voluntary intake. With respect to water-soluble carbohydrate and fibre concentrations of the herbage, the lack of consistency between years are possibly due to the changes observed in the percentage of chicory present in the sward although it is recognised that this could also have been due to seasonal differences, which can also influence these parameters in forages [49]. The lack of difference in the ash content between the forage treatments was unexpected, given the reported higher mineral and trace element content of chicory [8], [13], [50]. However, forage quality, as shown by its chemical composition, was good for both treatments and, in summary, should not have restricted the voluntary intake capacity of the steers during this study.

### Animals

#### Performance

Previous research into beef animals grazing chicory, coupled with the findings of the benefits of this forage within sheep and deer production systems [5], [14], [17]–[18], provided some evidence for a hypothesis that the inclusion of chicory in pasture swards could improve the production performance of grazing beef animals. In a short-term leader-follower experiment with calves and bulls grazing chicory at two different herbage allowances (representing mid-reproductive and mature pasture growth stages), beef animals were found to preferentially graze the leaf before the stem and the provision of chicory at a vegetative/high leaf stage achieved calf live-weight gains of 0.60 kg per day [21]. The authors concluded that further studies were needed to determine the longer-term production response of beef cattle when grazed on chicory maintained in vegetative state. Research conducted into the effects of two different herbage allowances for beef grazing chicory on bite rate [22] and found high intake rates with cattle consuming 3.6 kg per h when provided with chicory at an allowance of 5.2 kg per cow per hour and concluded that chicory could be used in beef systems for livestock having high nutrient and DM intake requirements.

Whilst there have been no other studies comparing the effects of chicory/perennial ryegrass to perennial ryegrass pastures, the findings in the current study, showing that the live-weight gain of steers grazing the chicory/ryegrass was comparable to ryegrass swards, confirm the observations of studies conducted by [23]. In an experiment conducted in the southeast of the United States, chicory was found to produce live-weight gains in beef cattle that were comparable to those for beef grazing pastures sown with an annual ryegrass (*Lolium multiflorum*) [23]. It is possible that the lack of difference between treatments, as observed in the current study and in [23], was due to the average daily live-weight gains of the animals in both experiments being relatively high. This suggestion is supported by recent findings [24] reporting similar live-weight gains for steers grazing chicory as reported here (1.13 kg day^−1^) but showed that this production response was higher when compared with cattle grazing other forages in the same study (0.56, 0.76 and 0.88 kg day^−1^ for pearl millet (*Pennisetum glacum*), bermudagrass (*Cynodon dactylon*) and cowpea (*Vigna unguiculata*), respectively), with the exception of lucerne (*Medicago sativa*) which produced higher live-weight gains (1.28 kg day^−1^). Hence, the live-weight gain values reported in the current study are regarded as a good response for beef cattle grazing temperate pastures during the main growing season [51], [52], thus reducing the chances of obtaining an improvement in production and warranting a conclusion of there being no detrimental effects to the inclusion of chicory within pastures used for finishing beef systems.

Furthermore, in practical farm situations, chicory is typically used in one of two ways: either as a medium-term ley in a mix with grass and clovers or as a pure sward for a short-term lamb or deer-finishing crop, with most of the research showing higher productivity responses for sheep and deer being conducted using these specialist short-term forage chicory crops. However, due to the long-term nature of forage-based beef finishing systems, in the current study the chicory was sown within a mix with perennial ryegrass to make the current experiment applicable to a practical farming system. Clovers were excluded from the mixture to reduce any interactions if clover levels differed between treatments. Using chicory within a ryegrass mix was also more likely to reduce the risk of any potential effects of a wet grazing season on either DM intakes or sward damage whilst a two-year experiment was completed. However, it is possible that this reduced the potential production response that can be gained from the inclusion of chicory in the diet of beef steers and further studies into the effects of pure chicory swards grazed late season or in the second season only are needed to investigate this further.

#### Parasite status

The finding in this study that beef steers grazing chicory/ryegrass swards did not have lower FEC than steers grazing ryegrass is in agreement with some previous research on the effects of chicory on FEC in sheep on chicory pastures in New Zealand and in the UK [18], [53] but is in disagreement with other previous findings [15]. One possible reason for the disparity in the literature on the effects of chicory on FEC is due to the finding that chicory may only reduce the abomasal helminth parasite species as shown in lambs grazing chicory [18]. Furthermore, faecal egg counts need to be treated with caution when used as an estimate of clinical effects as it can be affected by many variables and is only an indication of the number of reproductively–active female parasites and does not account for differences in the fecundity of different parasite species or the number of immature parasites [54]. To help overcome this, parasitism in the beef steers in the current study was determined by measuring three variables (faecal egg counts, plasma pepsinogen and O. ostertagi antibodies), as multiple parameters have been found to provide a more accurate representation of the parasite status of an animal than FEC alone [55]. However, contrary to the research reporting that lambs grazing chicory had reduced the pepsinogen concentrations in their blood [15], no differences were found in pepsinogen, or indeed antibodies to O. ostertagia, between forage treatments for cattle in the present study. Whilst recognising the limitation of faecal cultures to provide an insight into the predominant parasite species within the live animal [56], previous studies have confirmed that chicory does not alter the hatchability of helminth parasite eggs in ovine faeces [57]. Therefore, in the current study, faecal cultures were conducted on faecal samples from the beef steers and O. ostertagi, which resides in the abomasum, was found as one of two dominant species, confirming that the animals were infected with abomasal parasites. The measurement of serum pepsinogen concentrations has been recommended as a specific tool in the diagnosis of ostertagiosis [58]. The serum pepsinogen concentrations further confirm the presence of abomasal pathology associated with a low to moderate level of O. ostertagi with the steers having tyrosine levels of approximately 2 U, indicative of subclinical disease. Concentrations with a group mean above 5 U are observed in clinical ostertagiosis [58]–[59].

The lack of effects of the inclusion of chicory in swards on the parasites of steers in this study may be simply because there are none in cattle, but the experimental design precluded any direct perturbations of parasite populations between treatment groups as this would have confounded the primary objectives of measuring effects of performance and carcass quality. In addition, the parameters that were measured may have not been sensitive enough to detect subtle differences in parasitism between the treatment groups. For example, the fact that the parasite levels were only monitored between anthelmintic treatments may have limited the ability to detect differences. Furthermore, the use of a mixed chicory/ryegrass sward instead of a pure chicory sward, as used in other experiments, may have removed one of the other underlying mechanisms by which this forage may be reducing helminth parasites in sheep. Studies have shown that the micro-environment within a chicory sward can reduce the total number of immature larvae of helminth parasites that hatch, survive and migrate within pure chicory swards in both New Zealand and the UK [60]–[61]. This is not withstanding that it is recognised that it is difficult to draw comparisons between cattle and sheep, given species differences in parasite species, grazing habits, digestive efficiencies, intakes, etc. [62]. In conclusion, whilst the data from this study provide interesting observations, further (short-term) studies comparing beef steers, naturally-infected with internal parasites and grazing either pure chicory swards or pure ryegrass swards are needed to fully elucidate any effects of chicory on internal parasites in beef systems.

#### Carcass characteristics

There have been no studies into the effect of chicory on the carcass characteristics of beef steers as investigated in this current study. However, similar work conducted in lambs showed, in contrast to the findings in the current study, that finishing lambs on chicory resulted in carcasses with a better killing out percentage and carcase conformation score [19]. The reason for the disparity in these findings compared to the current study may again be due to differences between ruminant species or that the lambs experiment used pure chicory compared to a grass/clover control sward. Overall, in the present study, the beef cattle had an average classification score of 3R, which translates into about 19% of separable fat in the carcass [38]. This means that all carcasses were correct for the retail market (approximately forty per cent of carcasses produced in the UK are graded as R and forty per cent are graded as U), and that there were no detrimental effects of including chicory in grazing swards on the carcass characteristics of finished beef steers.

## Conclusions

The results of this study show that the inclusion of chicory in the diet of grazing beef steers did not alter their performance, faecal egg counts, blood indicators of helminth parasitism or carcass characteristics when compared with beef steers grazing ryegrass only swards. Short-term studies comparing beef steers finished on either pure vegetative chicory or ryegrass swards are needed to determine the potential benefits of chicory within beef finishing systems. Further short-term studies comparing beef steers naturally-infected with internal parasites and grazing either pure chicory or ryegrass swards are needed to elucidate the effects of chicory on internal helminth parasites in cattle.
